# Vaccination Targeting a Surface Sialidase of *P. acnes:* Implication for New Treatment of Acne Vulgaris

**DOI:** 10.1371/journal.pone.0001551

**Published:** 2008-02-06

**Authors:** Teruaki Nakatsuji, Yu-Tsueng Liu, Cheng-Po Huang, Richard L. Gallo, Chun-Ming Huang

**Affiliations:** 1 Division of Dermatology, Department of Medicine, University of California San Diego, San Diego, California, United States of America; 2 Veterans Affairs (VA) San Diego Healthcare Center, San Diego, California, United States of America; 3 Moores Cancer Center, University of California San Diego, San Diego, California, United States of America; 4 La Jolla Institute for Molecular Medicine, San Diego, California, United States of America; Max Planck Institute for Infection Biology, Germany

## Abstract

**Background:**

Acne vulgaris afflicts more than fifty million people in the United State and the severity of this disorder is associated with the immune response to *Propionibacterium acnes (P. acnes)*. Systemic therapies for acne target *P. acnes* using antibiotics, or target the follicle with retinoids such as isotretinoin. The latter systemic treatment is highly effective but also carries a risk of side effects including immune imbalance, hyperlipidemia, and teratogenicity. Despite substantial research into potential new therapies for this common disease, vaccines against acne vulgaris are not yet available.

**Methods and Findings:**

Here we create an acne vaccine targeting a cell wall-anchored sialidase of *P. acnes.* The importance of sialidase to disease pathogenesis is shown by treatment of a human sebocyte cell line with recombinant sialidase that increased susceptibility to *P. acnes* cytotoxicity and adhesion. Mice immunized with sialidase elicit a detectable antibody; the anti-sialidase serum effectively neutralized the cytotoxicity of *P. acnes in vitro* and *P. acnes*-induced interleukin-8 (IL-8) production in human sebocytes. Furthermore, the sialidase-immunized mice provided protective immunity against *P. acnes in vivo* as this treatment blocked an increase in ear thickness and release of pro-inflammatory macrophage inflammatory protein (MIP-2) cytokine.

**Conclusions:**

Results indicated that acne vaccines open novel therapeutic avenues for acne vulgaris and other *P. acnes*-associated diseases.

## Introduction

*P. acnes*, a gram-positive bacterium, is strongly associated with acne vulgaris. Isotretinoin, 13-cis-retinoic acid, is a vitamin A-derived retinoid that has been widely prescribed for systemic treatment of severe acne. However, the teratogenicity of isotretinoin is well documented [1], [2]. Although isotretinoin was first approved in the United State in 1982 for treating severe acne, its use has become tightly regulated and it is not appropriate for most acne patients. Other therapies, such as systemic antibiotic treatments, also have limitations. These therapies may kill skin bacteria non-specifically, impacting the homeostasis of resident dermal microflora [3], [4]. In addition, oral antibiotics contain the risk of harming the intestinal microflora. The presence of *P. acnes* prevents colonization by more harmful bacteria [*Staphylococcus aureus* (*S. aureus)* and *Acinetobacter baumannii*, and it has been reported that *P. acnes* can transfer anti-bacterial resistance to other bacteria within the resident skin microflora when systemic antibiotic therapy is used [3]. Recently, antibiotic-resistant *P. acnes* has been found with the use of antibiotics [5]. Currently available topical treatments for acne lesions, including drugs, are palliative, requiring a sustained treatment regiment. When these therapies are discontinued, acne inevitably recurs. Acne vulgaris is a multi-factorial disease associated with *P. acnes* infection, hormone regulation and immune responses [1], [3]. The inflammatory stage of acne vulgaris is usually the greatest concern to patients, as the lesions produced may lead to scarring and adverse psychological effects. Therefore, vaccines that suppress *P. acnes*-induced inflammation and pathogenesis, while minimizing the risk of altering the homeostasis of hormones and microflora, could be clinically valuable.

The complete genome of *P. acnes* has been sequenced [6]. Genomic data has revealed many gene encoded virulence factors, including sialidase, that are involved in degrading host tissue and inducing inflammation [7]. These virulence factors, which are either secreted from *P. acnes* or anchored in its cell wall, stimulate adjacent sebocytes and keratinocytes, triggering acne inflammation. Sialidases of *P. acnes* can cleave sialoglycoconjugates to obtain sialic acids for use as carbon and energy sources [6]. Sialidase has been used previously as a vaccine target for several diseases, including influenza and bacterial pneumonia [8], [9]. Thus, we chose a *P. acnes* surface sialidase (accession number: gi|50843035) containing an LPXTG cell wall-anchoring motif in the C-terminal domain [6], [7] as a target for acne vaccine development. Our data demonstrated that sialidase-immunized mice demonstrated decreased *P. acnes*-induced ear swelling and reduced production of the pro-inflammatory cytokine MIP-2, providing a rational design of acne vaccines that may offer a new treatment for acne vulgaris and other *P. acnes*-associated diseases.

## Methods

### Culture of *P. acnes*

*P. acnes* (ATCC® 6919) was cultured on Brucella broth agar, supplemented with 5% (v/v) defibrinated sheep blood, vitamin K, and hemin, under anaerobic conditions using Gas-Pak (BD Biosciences, San Jose, CA) at 37°C. A single colony was inoculated in Reinforced Clostridium Medium (Oxford, Hampshire, England) and cultured at 37°C until logarithmic growth phase. Bacterial pellets were harvested by centrifugation at 5,000 g for 10 min.

### Molecular Cloning and Expression of Recombinant Sialidase

A polymerase chain reaction (PCR) product encoding a putative mature *P. acnes* cell wall anchored sialidase (accession number: gi|50843035) was generated using gene-specific primers designed based on the *P. acnes* complete genome sequence. The forward PCR primer (5′- TAAGGCCTCTGTCGACTCAGGCAGGGCTCCGGCCCCAGATGC-3′) included 16 nucleotides containing a Sal I site (GTCGAC) to match the end of the In-Fusion Ready pEcoli-Nterm 6xHN vector (Clontech Laboratories, Inc., Mountain View, CA), and 26 nucleotides encoding the N-terminal of sialidase. The reverse PCR primer (5′- CAGAATTCGCAAGCTTGTCTCCTGTGTGCGGCAAACTAG-3′) consisted of 16 nucleotides containing a Hind III site (AAGCTT) to match the end of the vector and 23 nucleotides encoding the C-terminal of the protein. PCR was performed using the forward and reverse primers and *P. acnes* genomic DNA as template. The amplified fragment was inserted into an In-Fusion Ready pEcoli-Nterm expression plasmid. Competent cells (*E. coli, BL21 (DE3),* Invitrogen, Carlsbad, CA) were transformed with this plasmid, selected on Luria-Bertani (LB)-plates containing ampicillin (50 µg/ml) and an isolated colony was cultured overnight at 37°C with gentle shaking. An aliquot of the overnight culture was diluted 1∶20 with LB-medium and incubated at 37°C until reaching OD_600_ = 0.7. Isopropyl-ß-D-thiogalactoside (IPTG) (1 mM) was added into culture for 4 h to induce protein synthesis. Bacteria were harvested by centrifugation, rinsed with phosphate buffered saline (PBS), and suspended in 1/10 volume PBS. The bacteria were disrupted by sonication on ice for 1 min and lysed by centrifuging at 3,000 g for 30 min. The pellet was washed with PBS and dissolved in 50 mM sodium phosphate buffer containing 6 M guanidine HCl and 300 mM NaCl. The expressed protein possessing 6x HN tag was purified in denaturing condition with a TALON Express Purification Kit (Clontech Laboratories, Inc., Mountain View, CA). Subsequently, the purified protein was dialyzed against H_2_O, and then lyophilized. The lyophilized protein was dissolved in ethylene glycol (1 mg/1.2 ml), and then stirred in a refolding buffer (10 ml, 250 mM Tris-HCl buffer, pH 8.4, containing 5 mM cysteine, 0.5 mM cystine, and 1.5 M urea) at 4°C overnight. The refolded protein was dialyzed against PBS (pH 6.0), and concentrated. 10% SDS-polyacrylamide gel electrophoresis (SDS-PAGE) and subsequent gel staining with Coomassie blue were used for detection of protein expression.

### Protein Identification via NanoLC- LTQ MS/MS Analysis

In-gel digestion with trypsin and protein identification via NanoLC-LTQ mass spectrometry (MS) analysis were performed essentially as described previously [10]. The automated NanoLC-LTQ MS/MS setup consisted of an Eksigent Nano 2D LC system, a switch valve, a C18 trap column (Agilent, Santa Clara, CA), and a capillary reversed phased column (10 cm in length, 75 µm i.d.) packed with 5 µm, C18 AQUASIL resin with an integral spray tip (Picofrit, 15 µm tip, New Objective, Woburn, MA). A reversed-phase LC directly coupled to a LTQ ion trap mass spectrometer (Thermo Electron, Waltham, MA) was run using a linear gradient elution from buffer A (H_2_O plus 0.1% formic acid) to 50% buffer A plus 50% buffer B (acetonitrile plus 0.1% formic acid) in 100 min. The instruments were operated in the data dependent mode. Data on the four strongest ions above an intensity of 2×10^5^ were collected with dynamic exclusion enabled and the collision energy set at 35%. Large-scale MS/MS spectra were extracted using default value by Bioworks® 3.2 (Thermo Scientific, San Jose, CA). Charge state deconvolution and deisotoping were not performed. All MS/MS spectra were analyzed using in-house Sorcerer™ 2 system with SEQUEST (v.27, rev. 11) as the search program for protein identification. SEQUEST was set up to search the target-decoy ipi.MOUSE.v3.14 database containing protein sequences in both forward and reverse orientations (68627 entries in each orientation) using trypsin as the digestion enzyme with the allowance of up to five missed cleavages. The false positive rates were roughly determined by doubling the ratio between the number of decoy hits and the total number of hits. SEQUEST was searched with a fragment ion mass tolerance of 0.5 Da and a parent ion tolerance of 1.0 Da.

### Sebocyte Culture and Cell Death Determination

An immortalized human sebocyte line SZ95 was obtained as a gift from Dr. Zouboulis CC in the Departments of Dermatology and Immunology, Dessau Medical Center, Dessau, Germany. Sebocytes were cultured in Sebomed Basal medium (Biochrom, Berlin, Germany), supplemented with 5 ng/ml human recombinant epidermal growth factor (Sigma, St. Louis, MO), 10% (v/v) heat-inactivated fetal bovine serum, at 37°C under atmosphere of 5% (v/v) CO_2_ in air. For determination of sialidase activity and the effect of sialidase on the sebocytes' susceptibility to *P. acnes* infection, sebocytes (1.5×10^5^) were incubated in a 96-well micro plate with 10 µg/ml of recombinant sialidase or green fluorescent protein (GFP) in the medium with the pH adjusted to 6.0 for 2 h. Incubation with the same volume of PBS served as a control. The sebocytes treated with sialidase or GFP were then co-cultured with *P. acnes* [multiplicity of infection (MOI) 1∶10/cell: bacteria] for 18 h. After the co-culture, unbound bacteria were extensively washed three times with PBS. Dead sebocytes stained with trypan blue were counted on a hemocytometer. The colony forming unit (CFU) of *P. acnes* incorporated with sebocytes was determined by spreading serial dilutions of aliquots of trypsinized sebocyte suspension in 0.01% (w/v) Triton-X on agar plates to quantify CFU/cell. The adherence of *P. acnes* to sebocytes was visualized by staining with Accustain Gram stain kit (Sigma, St. Louis, MO).

### Flow Cytometry

Sebocytes (1.5×10^6^) were incubated with recombinant sialidase (10 µg/ml) at pH 6.0 for 2 h. The sebocytes were washed with PBS three times and fixed with 1% formaldehyde in PBS for 5 min at room temperature. After washing, the cells were incubated at 37°C for 15 min with 10 µg/ml of biotinylated MAA lectin I (Vector Laboratories, Burlingame, CA), which was prepared with 1% (w/v) bovine serum albumin (BSA) in PBS. The bound biotin was reacted with 1.5 µg/ml of a streptavidin-streptavidin-fluorescein isothiocyanate (FITC) conjugate (Jackson immunoresearch, West Grove, PA), which was incubated in 1% (w/v) BSA in PBS at 37°C for 10 min. After trypsinizing, the fluorescence intensities of the cells were analyzed with a flow cytometer (FACSCalibur, BD Biosciences, San Jose, CA).

### Vaccination and Antibody Detection

Female ICR mice approximately 3-months-old (Harlan, Indianapolis, IN) were used for vaccination. Recombinant sialidase or GFP was dissolved in PBS and mixed with an equal volume of complete or incomplete Freund's adjuvant. For the first vaccination, 50 µg of recombinant protein in complete Freund's adjuvant was injected subcutaneously into the dorsal skin. Two weeks later, 50 µg of recombinant protein in incomplete Freund's adjuvant was intraperitoneally injected for second boost. One week after the second boot, serum containing immunoglobulin G (IgG) antibody was harvested for western blot analysis. Serum was diluted 1∶10,000 for the reaction. To obtain antiserum against *P. acnes*, ICR mice were vaccinated intranasally with killed *P. acnes* (25 µl; 10^8^ CFU) for nine weeks (three boosts at three-week intervals). *P. acnes* was killed by heat at 65°C for 30 min or ultraviolet (UV) at 7,000 mJ/cm^2^. Anti-serum raised against killed *P. acnes* was collected one week after the third boost. Each group (n = 4) and each experiment was performed in triplicate. 10% SDS-PAGE was used for western blot analysis. All experiments using mice were conducted according to institutional guidelines.

### Immune Protection by A Sialidase-based Vaccine

Live *P. acnes* (20 µl; 10^7^ CFU) was subcutaneously injected in the central portion of the left ear of sialidase- or GFP- vaccinated mice. 20 µl of PBS was injected into the right ear as a control. After injection, ear thickness was measured using a micro caliper (Mitutoyo, Aurora, IL) and recorded periodically until ear swelling had nearly subsided (71 days). The *P. acnes*-induced change in ear thickness was calculated as % of that in PBS-injected ears. To assess the effect of vaccination on *P. acnes* growth, ears injected with PBS or live *P. acnes* in sialidase- or GFP-immunized mice were excised and homogenized at eight days after injection. *P. acnes* from homogenized ears were grown on agar plates for CFU counting.

### Tissue Chamber and Pro-inflammatory Cytokine Detection

ICR mice were anesthetized with 10 mg of ketamine and 1.5 mg of xylazine per 100 g of body weight. The tissue chamber (internal and external diameters, 1.5 and 3.0 mm, respectively) consisted of closed ploytetrafluoroethylene Teflon cylinders with 12 regularly spaced 0.1 mm holes. The tissue chamber was sterilized by soaking in 70% ethanol overnight. The sterile tissue chamber was then implanted subcutaneously under abdominal skin and maintained in the mice for 7 days to ensure the chamber was fully integrated with the subcutaneous environment. For histological observation, an implanted tissue chamber was cross-sectioned, stained with haematoxylin and eosin (H&E) (Sigma diagnostics, St. Louis, MO), and viewed on a Zeiss Axioskop2 plus microscope (Carl Zeiss, Thornwood, NY). Fluids in the tissue chamber were drawn by pecutaneous aspiration before and after *P. acnes* (20 µl; 10^7^ CFU) injection for three days. The concentrations of MIP-2 and TNF-α in the tissue chamber fluid were determined with sandwich enzyme-linked immunosorbent assay (ELISA) kits (R & D Systems Inc., Minneapolis, MN) according to the provided protocols.

### *In Vitro* Neutralization Assays

For *in vitro* neutralization assays, *P. acnes* (2×10^6 ^CFU) was pre-treated with 2.5 % (v/v) of anti-sialidase or -GFP serum in the medium at 37°C for 2 h before being added to sebocyte (2×10^5^) cultures for 18 h. Complement in the serum was deactivated by heating at 56°C for 30 min. Sebocyte death induced by *P. acnes* was determined with *p*-Nitrophenyl phosphate disodium (*p*NPP) (Pierce, Rockford, IL) according to the method of Martin and Clynes [11]. The sebocytes were washed with PBS three times and reacted with *p*NPP for 1 h at 37°C. The absorbance of the *p*NPP reaction was measured at OD_405 _nm. As a baseline, sebocytes killed with 0.1% (v/v) Triton-X were also assayed. Sebocyte death induced by *P. acnes* cytotoxicity was calculated as (the OD_405 _difference without and with *P. acnes* treatment)/(the OD_405 _difference without *P. acnes* and with Triton-X treatment)×100 %. For IL-8 detection, *P. acnes* (1.5×10^8^ CFU) pre-treated with anti-sialidase or anti-GFP sera was added into sebocyte (3×10^6^) cultures for 8 h. After centrifuging to remove bacteria, the concentrations of IL-8 in the culture medium were determined by ELISA assays (R & D Systems Inc.).

### Statistical Analyses

Data are presented as mean±standard error (SE). The Student *t*-test was used to assess the significance of independent experiments. The criterion *p*<0.05 was used to determine statistical significance.

## Results

### Sialidase Expression and Mass Spectrometric Identity

**Figure 1:**
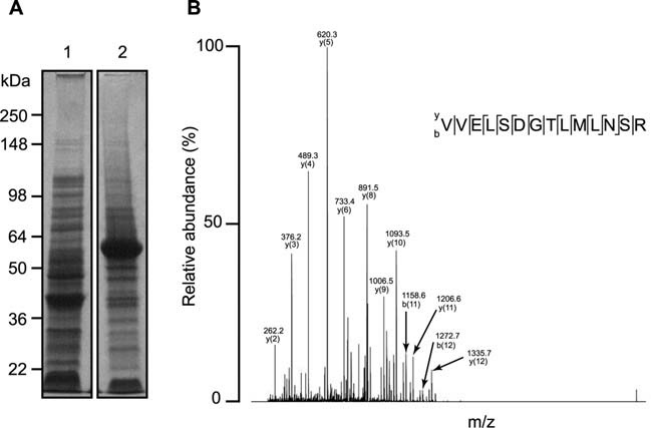
Expression and Purification of A Cell Wall Anchored Sialidase. (A) A vector encoding a cell wall anchored sialidase (accession # gi|50843035) was constructed by inserting a PCR amplified full sialidase gene into the pEcoli-Nterm 6xHN vector (Clontech Laboratories, Inc., Mountain View, CA) at the SaII and HindIII restriction sites. Specific primers including the sense (5′- ATGACTTTGACCACGAAACTGAGCG-3′) and anti-sense primers (5′-TCAGGCAGGGCTCCGGCCCCAGATGC-3′) were designed to clone sialidase from *P. acnes* (ATCC 6919). The vector, which contains T7/LacO promoter, is derived from the pET system developed by William Studier and colleagues to achieve exceptionally high levels of protein expression in *E. coli*. Sialidase (arrow) was expressed in *E. coli* in the absence (lane 1) or presence (lane 2) of (1 mM) IPTG. After IPTG induction, sialidase was successfully expressed in *E. coli* and shown at about 53.1 kDa on a 10 % SDS-PAGE (arrowhead). (B) Purified sialidase (arrow) was obtained via In-Fusion Ready TALON Express Bacterial Expression and Purification kit (Clontech Laboratories, Inc., Mountain View, CA). (C) The expression and purity of sialidase were confirmed by Nano-LTQ MS/MS mass spectrometry (Thermo Electron Corp. Waltham, MA). A sequenced internal peptide (VVELSDTLMLNSR) of sialidase was presented.

A gene encoding sialidase was amplified by PCR using specific primers from template DNA prepared from *P. acnes*. The PCR products were inserted into a pEcoli-Nterm 6xHN plasmid and expressed in *E. coli* [*E. coli BL21 (DE3)*]. After IPTG induction, over-expressed sialidase-6xNH fusion protein from *E. coli* was detected by SDS-PAGE with Coomassie blue staining at approximately 53.1 kDa molecular weight (Figure 1A). The sialidase (Figure 1B) was purified using a TALON resin column. The sialidase expression was confirmed by NanoLC-LTQ MS/MS mass spectrometric sequencing after in-gel trypsin digestion (Figure 1C). Nineteen internal peptides (data not shown) derived from sialidase were fully sequenced by NanoLC-LTQ MS/MS mass spectrometry, matching well with those from *P. acnes* sialidase (accession number: gi|50843035). An internal peptide (VVELSDGTLMLNSR; 316–329 amino acid residues) of sialidase is presented (Figure 1C), validating the expression of recombinant sialidase.

### The Susceptibility of Sebocytes to *P. acnes* after Sialidase Treatments

**Figure 2:**
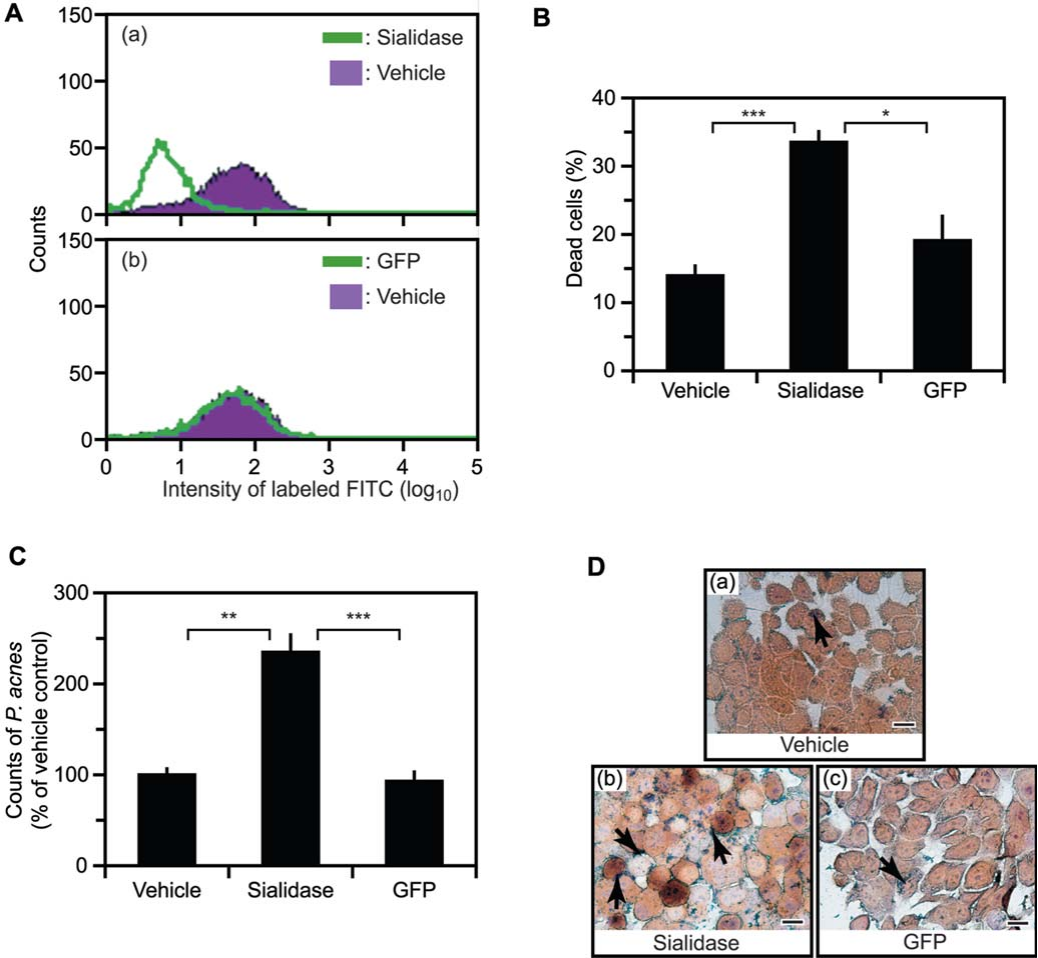
Removal of Sialic Acids from Human Sebocytes by Sialidase Increases Their Susceptibility to *P. acnes.* (A) Sialic acids on the cell surface of immortalized human sebocytes (SZ95) were detected by their reaction with biotinylated MAA lectin I (10 µg/ml) and streptavidin-FITC conjugate. FITC-fluorescence intensity was measured by flow cytomtery (FACSCalibur, BD Biosciences, San Jose, CA), reflecting the quantity of sialic acid. The sebocytes were pre-treated with 10 µg/ml of purified recombinant sialidase (green, a), GFP (green, b) or an equal volume of PBS (vehicle) (purple) at pH 6 for 2 h. The decrease in FITC-fluorescence intensity in sialidase-treated sebocytes validated the enzymatic activity of the purified sialidase. (B) After pre-treatment with sialidase, sebocytes were co-cultured with *P. acnes* (5×10^7^ CFU/5×10^6^ cells) for 18 h. *P. acnes*-induced cell death in PBS (vehicle)-, sialidase- or GFP-pretreated sebocytes was assessed by trypan blue. Cell death is presented as the % of dead cells compared to all cultured cells. (C) After washing out unbound *P. acnes*, the number of *P. acnes* adhered to sebocytes was calculated by spreading Triton-X (0.01%) lysed sebocytes on agar plates to quantify CFU/cell. (D) The CFU/cell in vehicle-pretreated sebocytes was defined as 100%. Adherence of *P. acnes* (arrows) to vehicle (a)-, sialidase (b)- or GFP (c)-treated sebocytes was visualized by staining with the Accustain Gram stain kit. Pre-treatment with sialidase significantly increased the adhesion of *P. acnes* to sebocytes. Shown are representative results of three independent experiments. Error bars are mean±SE (*P*<0.01*, *P*<0.001**, *P*<0.0005*** by Student's t-test). Bar: 10 µm.

Real-time PCR revealed that the *P. acnes* expressed sialidase (Text S1 and Figure S1). The experiment was conducted by using a triacylglycerol lipase of *P. acnes* as a positive control. To determine its enzymatic activity, purified sialidase (10 µg/ml) was added to human SZ95 sebocyte cultures for 2 h to remove the sialic acids on the surface of sebocytes. Surface sialic acids were quantified by flow cytometry (FACSCalibur, BD Biosciences, San Jose, CA) using biotinylated *Maackia Amurensis* (MAA) lectin I and FITC conjugate. The fluorescence of MAA labeled-sialic acids in sialidase-treated sebocytes was reduced by approximately 69% (Figure 2A, a), whereas the fluorescence in control sebocytes treated with GFP was unchanged (Figure 2A, b). These data indicate that our purified recombinant sialidase enzymatically cleaves sialoglycoconjugates, releasing sialic acids. Treatment of purified sialidase for 2 h did not influence the cell viability of sebocytes (data not shown). After treatment with sialidase, or controls, for 2 h, sebocytes (5×10^6^ cells) were exposed to live *P. acnes* (5×10^7^ CUF) overnight. Live *P. acnes* induced approximately 15∼20% cell death in PBS (vehicle)- or GFP-treated sebocytes, whereas sialidase-treated sebocyte cell death was significantly higher, at 33.5±1.8 % (Figure 2B), suggesting that sialidase treatment increases the susceptibility of sebocytes to *P. acnes*. It has been demonstrated that incubation of human buccal epithelial cells with sialidase greatly augments *Pseudomonas aeruginosa* adherence [12], leading us to examine the adherence of *P. acnes* to sialidase-treated sebocytes. Pre-treatment with sialidase, but not GFP (10 µg/ml for 2h), significantly increased the association of *P. acnes* with sebocytes (Figure 2, D). Accustain Gram stains also clearly indicated that the number of *P. acnes* interacting with sebocytes was elevated once sebocyte surface sialic acids were removed (Figure 2D, a–c). These data, combined with the fact that sialidase is a surface protein carrying a cell wall-anchoring LPXTG motif, make it a potentially valuable target for creating vaccines against *P. acnes*-associated diseases, such as acne vulgaris.

### Immunogenicity in Mice Vaccinated with Recombinant Sialidase

**Figure 3:**
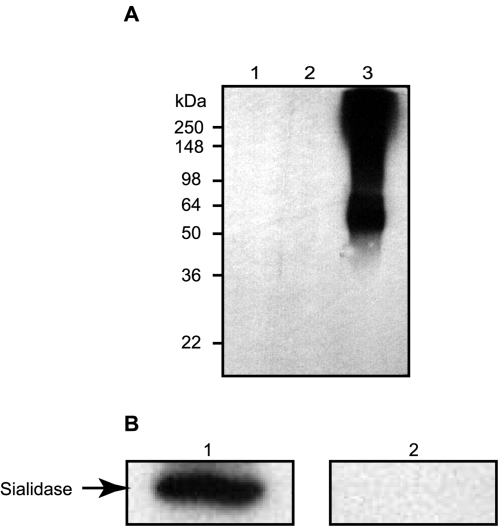
Mice Immunized with Recombinant Sialidase and Freund (in)complete Adjuvants Produce Sialidase Specific Antibodies. (A) ICR mice were vaccinated with heat-killed *P. acnes* for nine weeks (three boosts at a three-week interval). Serum (1: 500 dilution), harvested one week after the third boost, was reacted to recombinant sialidase (1 µg; lane 1), GFP (1 µg, lane 2), and *P. acnes* lysates (7 µg, lane 3) that had been run on a 10% SDS-PAGE. The 6x HN tag of recombinant sialidase was removed by enterokinase before loading into a SDS-PAGE. Although heat-killed *P. acnes*-vaccinated mice produced anti-sera against several *P. acnes* proteins (>50 kDa) (lane 3), sialidase specific antibodies were not detected (lane 3). (B) ICR mice were then vaccinated with a recombinant sialidase (lane 1) or GFP (lane 2) using Freund/(in)complete adjuvants. For the first vaccination, mice were subcutaneously inoculated with 50 µg of sialidase or GFP, which were emulsified with a complete Freund adjuvant. Two weeks later, the second vaccination was delivered via intramuscular injection with the same amount of antigen mixed well with incomplete Freund adjuvant. Anti-sialidase antibodies were detected by western blot one week after the second vaccination. 1 µg of recombinant sialidase or GFP were separated via 10 % SDS-PAGE, transferred to a PVDF membrane and reacted with mouse sera (10,000-fold dilution). Sialidase antibodies were provoked when mice were immunized with recombinant sialidase, but not with GFP. Data is representative of three separate experiments with similar results.

To assess the immunogenicity of sialidase, ICR mice were vaccinated with heat killed *P. acnes* for nine weeks. Recombinant sialidase, GFP and *P. acnes* lysates were subjected to western blot analysis. Many proteins with molecular weights greater than 50 kDa were immunoreactive with mouse serum obtained from the heat killed *P. acnes*-immunized mice (Figure 3A, lane 3), however, sialidase was not (Figure 3A, lane 1), indicating that mice immunized with whole organism *P. acnes* do not produce antibodies to sialidase. Similarly, recombinant sialidase was not immunoreactive to mouse serum obtained from UV-killed *P. acnes*-immunized mice (Figure S2), indicating that the undetected immunogenicity was not due to denaturation of sialidase during the heat treatment. We next vaccinated ICR mice with recombinant sialidase, or a GFP control, using Freund/(in)complete adjuvants. Antibody production in the serum of immunized mice was detected by western blot analysis four weeks after immunization (Figure 3B, lane 1). A strong band appearing at 53.1 kDa was visualized when purified sialidase was reacted with serum from sialidase-immunized mice, indicating that sialidase was immunogenic in mice vaccinated with recombinant sialidase. No immunoreactivity against sialidase was detectable in GFP-immunized mice (Figure 3B, lane 2).

### Protective Immunity Against *P. acnes* Challenge in Sialidase-vaccinated Mice

**Figure 4:**
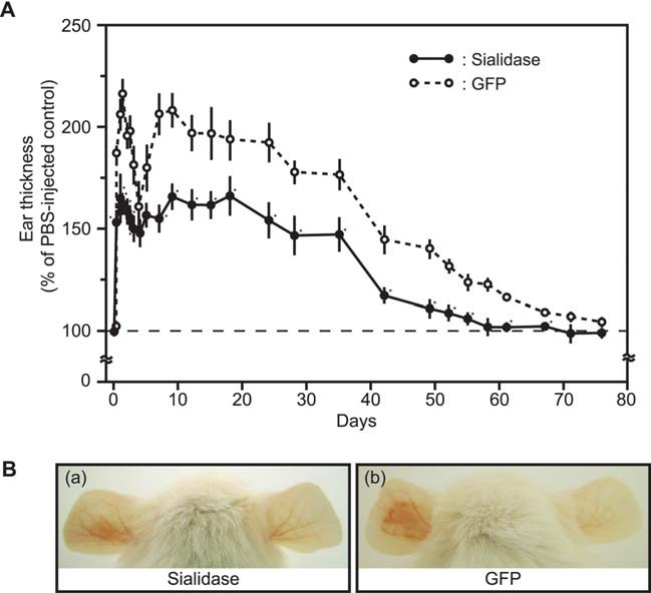
Immune Protection Conferred by A Sialidase-based Acne Vaccine. (A) The cytotoxicity of *P. acnes* was calculated as described in Methods and presented as mean±SE (*P*<0.0005** by Student's *t*-test). For assaying *in vivo* immune protection, ICR mice were immunized with recombinant sialidase or GFP using Freund (In)complete adjuvants (Figure 3). After confirming antibody production by western blot, live *P. acnes* (10^7^ CFU, 25 µl) were injected subcutaneously into the ears of sialidase- and GFP-immunized mice, with PBS (25 µl) as a control. Ear thickness was periodically measured for 71 days after injection and changes reported as % of ear thickness in PBS-injected ears. *P. acnes*-induced ear swelling was significantly suppressed in sialidase-immunized mice in comparison with GFP-immunized mice (*P*<0.05*), except for day 0, 4, 5 and 71. (B) Erythema was assessed in sialidase- (a) or GFP- (b) immunized mice 24 h after live *P. acnes* (left ears) or PBS (right ears) injection.

To asses immune protection *in vivo*, ICR mice immunized with recombinant proteins (sialidase or GFP) along with Freund/(in)complete adjuvants were challenged with live *P. acnes* (10^7^ CFU) three weeks after vaccination. One ear of each mouse was subcutaneously injected with 25 µl of *P. acnes* (10^7^ CFU) and the other ear was injected with 25 µl of PBS as a control. Injection of *P. acnes* induced ear swelling (Figure 4A) and redness (Figure 4B). Ear thickness was measured regularly for 71 days, revealing a biphasic ear-swelling pattern. Ear thickness in GFP-immunized mice rapidly increased more than two fold (215.8±7.7%) 32 h after *P. acnes* challenge, decreased, then rebounded four days after challenge. Ear swelling was significantly reduced in both phases (at 32 hours and 4 days post-challenge) by more than 50% when mice were immunized with sialidase (Figure 4A). Sialidase immunization also resulted in decreased erythema in ears challenged with *P. acnes* (Figure 4B). Ear swelling in GFP-immunized mice nearly subsided 71 days after *P. acnes* challenge, whereas sialidase-immunized mice were completely recovered 58 days after challenge. These results indicate that sialidase-immunized mice suppressed *P. acnes*-induced ear inflammation.

### Detection of Pro-inflammatory Cytokines in Implanted Tissue Chambers

**Figure 5:**
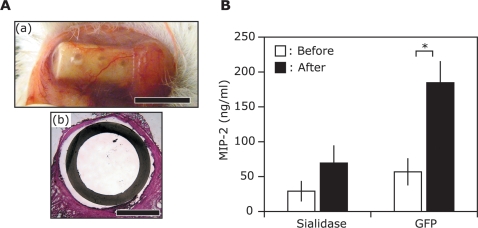
A Sialidase-based Acne Vaccine Suppresses Induction of the Pro-inflammatory Cytokine MIP-2 by *P. acnes.* (A) A tissue chamber (a) (internal and external diameters, 1.5 and 3 mm, respectively, length, 1 cm; internal volume, 80 µl) was implanted subcutaneously in the abdomen of sialidase- or GFP-immunized mice. The tissue chamber consisted of closed ploytetrafluoroethylene Teflon cylinders with 12 regularly spaced 0.1 mm holes. Bar: 1 cm. H&E staining of the cross-section of an implanted tissue chamber showed that the chamber was completely encapsulated by fibrotic tissue 7-days after implantation (b). Bar: 1.0 mm. *P. acnes* (10^7^ CFU, 20 µl) was injected and trapped in this encapsulated tissue chamber. (B) Tissue chamber fluid containing MIP-2 was drawn by pecutaneous aspiration before (open bar) and three days after (solid bar) *P. acnes* injection. Measurement of MIP-2 was carried out by sandwich ELISA that used the Quantikine M mouse MIP-2 set (R&D System, Minneapolis, MN). Vaccination with sialidase markedly suppressed the *P. acnes*-induced increase in MIP-2. Error bars represented mean±SE of five separate experiments (*P<0.005 by Student's *t*-test).

Induction of pro-inflammatory cytokines also plays a key role in the progression of acne vulgaris. To determine whether sialidase immunization alters the level of *P. acnes*-induced pro-inflammatory cytokines, we employed a tissue chamber model (Figure 5A) to collect pro-inflammatory cytokines *in vivo*. The tissue chamber model has been extensively characterized in mice [13], [14] and accurately mimics bacterial infections *in vivo*. A tissue chamber was implanted subcutaneously in the abdomen of ICR mice 7 days before *P. acnes* (10^7^ CFU) inoculation. Three days after *P. acnes* inoculation, tissue chamber fluids containing pro-inflammatory cytokines were drawn by percutaneous aspiration and the levels of MIP-2 (Figure 5B) and (tumor necrosis factor) TNF-α were measured by ELISA. In GFP-immunized mice, a significant increase in the level of MIP-2 was observed 3 days after *P. acnes* inoculation, while sialidase-immunized mice demonstrated 61% less induction. The level of TNF-α remained unchanged after *P. acnes* inoculation in both GFP- and sialidase-immunized mice (data not shown). These results suggest that a vaccine targeting sialidase effectively suppresses *P. acnes*-induced production of the pro-inflammatory cytokine MIP-2 in the mice.

### Neutralization of Cytotoxicity of *P. acnes* to Human Sebocytes with Anti-sialidase Serum: Implication for Acne Treatment

**Figure 6:**
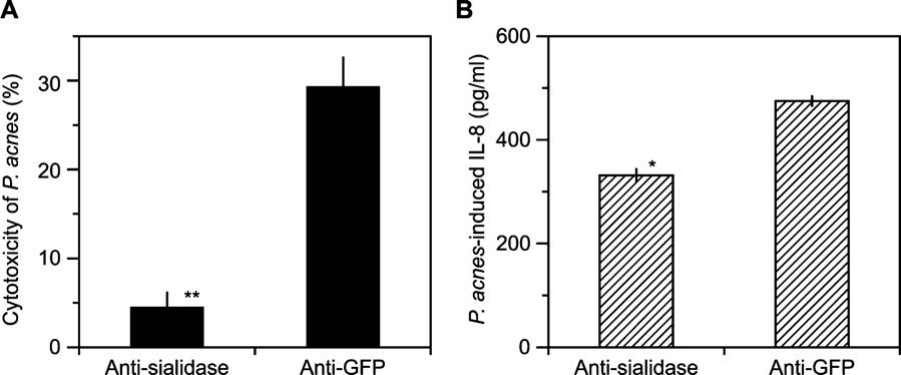
Neutralization with Anti-sialidase Serum Decreases *P. acnes*-induced Cell Death and IL-8 Production in Human Sebocytes. (A) For *in vitro* neutralization assays, *P. acnes* was pre-treated with 2.5% (v/v) anti-sialidase or GFP sera at 37°C for 2 h. Serum-pretreated *P. acnes* (2×10^6^ CFU) was then co-cultured with sebocytes (2×10^5^ cells) for 18 h. Sebocyte death induced by serum-pretreated *P. acnes* was detected by *p*NPP. The cytotoxicity of *P. acnes* was calculated as described in Methods and presented as mean±SE (n = 4, *P*<0.0005** by Student's *t*-test). (B) Serum-pretreated *P. acnes* (1.5×10^8^ CFU) was co-cultured with sebocytes (3×10^6^ cells) for 8 h. Measurement of IL-8 in the culture medium was carried out by ELISA assays using a Quantikine human IL-8 set (R&D System). The data is presented as mean±SE (n = 4, *P*<0.01* by Student's *t*-test).

In order to evaluate whether anti-sialidase serum was capable of neutralizing *P. acnes* cytotoxicity for sebocytes, *P. acnes* was pre-incubated with anti-sialidase serum at 37°C for 2 h prior to being added to sebocyte cultures. The CFU counting on agar plates showed that 2 h incubation with antisera did not impair the growth of *P. acnes* (data not shown). *P. acnes* pre-incubated with anti-GFP serum caused 29.3±3.4% of the sebocytes to undergo cell death, while pre-incubation with anti-sialidase serum dramatically reduced *P. acnes*-induced sebocyte death to 4.5±1.8% (Figure 6A). This data suggests that mice immunized with sialidase produce antibody that can neutralize the cytotoxicity of *P. acnes in vitro*. It has been shown that sebocytes expressed Toll-like receptor 2 (TLR2) and secreted cytokine IL-8 once they were stimulated with *P. acnes* [15]. In addition, expression of IL-8 was detectable in skin biopsies from patients with inflammatory acne vulgaris [16]. Thus, we next examine the capability of anti-sialidase serum in neutralizing *P. acnes*-induced IL-8 production in human sebocytes. Sebocytes stimulated with *P. acnes* that was pre-incubated with anti-GFP or anti-sialidase serum released IL-8 at the amount of 2.49±0.10 or 1.76±0.08 ng/ml, respectively (Figure 6B), indicating that anti-sialidase serum effectively suppresses *P. acnes*-induced IL-8 production in human sebocytes. Sebocytes within sebaceous glands are major target cells of *P. acnes* in patients with acne vulgaris [17]. Accordingly, generation of antibody against sialidase in acne patients may counteract the cytotoxicity of *P. acnes* to sebocytes and alleviate acne development.

## Discussions

It has been reported that treatment of human buccal epithelial cells with the sialidase considerably increased *Pseudomonas aeruginosa* adherence [18]. In addition, immunization with recombinant *Streptococcus pneumoniae* sialidase resulted in a significant reduction in pneumococcal colonization in the chinchilla model [19]. We demonstrated that the adherence of *P. acnes* to human sebocytes was augmented after removal of sialic acids from the cell surface. This result is in agreement with previous findings that sialidase is involved in the adhesion of pathogens to host cells [18], [20], and that treatment of host cells with sialidase changes their susceptibility to pathogens [19]. Adhesion process of bacteria occurs at the early stage of infection and is essential for its colonization, and in turn, colonization may be required for subsequent development of symptoms of diseases. Thus, vaccination targeting sialidase of *P. acnes* may be an efficient modality for the prevention of early infection of *P. acnes*.

Patients with acne lesions are likely to produce anti-*P. acnes* antibodies [21], however, acne lesions still recur in these patients. This suggests that patients infected with *P. acnes* may develop insufficient immunity to prevent subsequent *P. acnes* infection and acne recurrence. In this study, mice were immunized with either heat or UV killed *P. acnes* or recombinant sialidase. Our data has shown that sialidase is not immunogenic if vaccination with *P. acnes* is administered whereas sialidase becomes immunogenic when vaccination with recombinant protein is performed (Figure 3). An analysis of patients' sera by western blot assay recognized a 96 kDa antigenic component of *P. acnes* [22]. No reports demonstrated that sialidase is antigenic in the sera of acne patients [21], [22]. This result suggests that acne progression and recurrence could be effectively prevented if the antibody against sialidase of *P. acnes* can be robustly elicited in acne patients.

Inactivation of *P. acnes* has been used to create vaccines against acne vulgaris [23], [24]. Acnevac or autovaccines containing killed strains of *P. acnes* and/or *Staphylococci* have been tested in acne and normal healthy subjects [25]. Although these killed *P. acnes*-based vaccines showed a good effect on acne patients, their effect is based on the non-specific modulation of the immune system of patients. Furthermore, It has been shown that mice immunized with killed *P. acnes* demonstrate non-specific resistance to challenge with other microbes [26]. A single intraperitoneal injection of phenol-treated *P. acnes* into mice showed non-specific resistance against subsequent lethal doses of an intraperitoneal challenge of *Klebsiella pneumoniae, S. aureus,* and *Streptococcus pyogenes* (*S. pypgenes*) [26]. Recently, it has been shown that animals sensitized with *P. acnes* exhibit an increased susceptibility to *E. coli* lipopolysacharride (LPS) induced sepsis and [27] and liver failure [28]. The cell wall anchored sialidase presented in this article shares low identity with other surface sialidases in other pathogens [6]. Thus, acne vaccines utilizing a *P. acnes* specific sialidase instead of killed *P. acnes* as the immunogen may be more specific and reduce the chance of side effects. There are at least five sialidases [sialidase B (gi|50843035); cell wall anchored sialidase (gi|50843035); sialidase A precursor (gi|50842172); putative sialidase (gi|50843278) and sialidase-like protein (gi|50843043) in *P. acnes* genome. Although creation of *P. acnes* with sialidase mutation or over-expression may directly address the role of sialidase in the virulence of *P. acnes*, it may be a challenge to genetically alter all sialidases in individual *P. acnes*. However, developing a novel compound to block the enzyme activities of all sialidases in *P. acnes* may be of value [29].

Our data has demonstrated that a sialidase-based acne vaccine provided protective effects on *P. acnes*-induced ear inflammation (Figure 4). Ear thickness was measured regularly for 71 days, revealing a biphasic ear-swelling pattern. This is consistent with previous results demonstrating a biphasic change in the activity of the mouse reticuloendothelial system after intraperitoneal injection with phenol-treated *P. acnes* [26]. The biphasic pattern can be explained by two distinct stages: short-term/local (early phase) and long-term/systemic (late phase) immune stimulation. The fluctuation in the number of macrophages and other host cells reflected a biphasic pattern of *P. acnes* infection. The effect of sialidase immunization on *P. acnes* growth was also explored. Ears injected with PBS or live *P. acnes* (10^7^ CFU) in sialidase- or GFP-immunized mice were excised for homogenization eight days after bacterial challenge (data not shown). *P. acnes* from the homogenized ears were extracted and quantified on agar plates. The number of *P. acnes* in sialidase-immunized mice was not significantly different with that in GFP-immunized mice, indicating that sialidase immunization did not change the growth of *P. acnes*. Considering the data, the sialidase-based acne vaccine presented in this article may decrease *P. acnes*-induced inflammation without affecting the balance of body microflora.

Most animals including mice do not produce sufficient triglycerides in sebaceous glands to harbor *P. acnes* a fact that has encumbered the development of anti-acne vaccines and drugs targeting *P. acnes* infection [30]. Although Rhino mice with utricles that create larger follicles have been employed to determine compound comedogenicity [31], the genetic mutant mice cannot elicit antibodies against thymus-dependent antigens [32]. Thus, the use of Rhino mice as animal models may not be appropriate for vaccine evaluation. Rabbit ears have been utilized to determine compound comedogenicity of acne lesions [31]. However, the rabbit ear model has a lack of bacterial colonization and inflammation [33]. In addition, the use of rabbits may be inconvenient for vast vaccinations. A murine acne model measuring *P. acnes*-induced ear swelling and production of pro-inflammatory cytokines in tissue chambers may provide an alternative animal model for evaluating the potency of acne vaccines. Our data indicates that tissue chamber fluid contains various immune cells, including macrophages (CD11b^+^), neutrophils (Gr-1^+^), natural killer cells (CD49b^+^) and T cells (CD3^+^) (data not shown), suggesting an influx of immune cells into an implanted tissue chamber. In addition, IgG against *P. acnes* was detectable in tissue chamber fluids (data not shown) when tissue chambers were implanted into heat killed *P. acnes*-immunized mice. This result indicated that antibodies are able to migrate into tissue chambers to interact with injected *P. acnes*. By growing cells in a dermis-based cell-trapped system (DBCTS) and inserting into a tissue chamber, we successfully created a tissue microenvironment *in vivo* [34]. Thus, a bioengineering approach using a tissue chamber integrated with DBCTS may be able to create a humanized tissue microenvironment in animals to mimic the physiological structure of human hair follicles. The approach may eventually confer an animal model for evaluating of vaccines targeting hair follicles. We detected two important murine pro-inflammatory cytokines (MIP-2 and TNF-α) (Figure 5). It has been reported that recognition of *P. acnes* by TLR2 induces the activation of pro-inflammatory pathways [28]. *In vivo* priming of mice with *P. acnes* also results in elevated serum levels of TNF-α [28]. The lack of elevated TNF-α levels in tissue chamber fluid after *P. acnes* inoculation may reflect a difference in host response between the tissue microenvironment (tissue chamber) and the systemic environment (serum).

Overall, we present a novel vaccine targeting cell wall anchored sialidase of *P. acnes*. Antibodies against sialidase provoked in vaccinated mice effectively suppressed the *P. acnes*-induced inflammation (Figures 4 and 5) and neutralized the cytotoxicity of *P. acnes* to human sebocytes (Figure 6), implicating that the sialidase-based vaccine may have the potential for treatment of acne vulgaris, a most common skin disease affecting 85–100% of people at some point in their lives. In addition, the sialidase-based acne vaccine may be an alternative of the killed *P. acnes*-based vaccine that performed non-specifically and evoked many undesirable effects. Future directions include (i) establishing therapeutic acne vaccines that may benefit patients with severe acne and (ii) comparing the potency and side effects of sialidase-based vaccines with current medicines.

## Supporting Information

Figure S1 Quantitative analysis of the sialidase transcript in *P. acnes*. The gene expression of sialidase was determined by real-time quantitative PCR using specific primers as described in Methods. Total RNA isolated from anaerobically cultured *P. acnes* served as a template. The gene of triacylglycerol lipase known as a pathogenic factor of *P. acnes* was used as a positive control. A pGEM-T Easy Vector (Promega, Madison, WI) inserted with PCR products was performed to estimate the number of expressed genes. The level of gene expression of sialidase and triacylglycerol lipase was normalized to that of 16SrRNA gene.
(0.56 MB TIF)

Figure S2 Detection of immunogenicity of sialidase in mice vaccinated with UV-killed *P. acnes*. ICR mice were vaccinated with UV-killed *P. acnes* as described in Methods. Serum (1: 500 dilution) was reacted to recombinant sialidase (1 µg; lane 1), GFP (1 µg, lane 2), and *P. acnes* lysates (7 µg, lane 3) that had been run on a 10% SDS-PAGE. Sialidase and GFP were not immunoreactive to serum obtained from mice immunized with UV-killed *P. acnes*.
(0.33 MB TIF)

Text S1 (0.02 MB DOC)
