# Correction: Vaccination Targeting a Surface Sialidase of *P. acnes:* Implication for New Treatment of Acne Vulgaris

**DOI:** 10.1371/annotation/b54fb361-2909-49b8-8457-596b87d866ef

**Published:** 2008-08-19

**Authors:** Teruaki Nakatsuji, Yu-Tsueng Liu, Cheng-Po Huang, Christos C. Zouboulis, Richard L. Gallo, Chun-Ming Huang

Dr. Christos C. Zouboulis was not included in the author byline. He should be listed as the fourth author. Please view the corrected author list, affiliations, and citation here:

Teruaki Nakatsuji^1,2^, Yu-Tsueng Liu^3^, Cheng-Po Huang^2,3^, Christos C. Zouboulis^4^, Richard L. Gallo^1,2^, Chun-Ming Huang^1,2,3,5*^

1 Division of Dermatology, Department of Medicine, University of California San Diego, San Diego, California, United States of America 2 Veterans Affairs (VA) San Diego Healthcare Center, San Diego, California, United States of America 3 Moores Cancer Center, University of California San Diego, San Diego, California, United States of America 4 Departments of Dermatology, Venerology, Allergology, and Immunology, Dessau Medical Center, Dessau, Germany 5 La Jolla Institute for Molecular Medicine, San Diego, California, United States of America 

* To whom correspondence should be addressed. E-mail: chunming@ucsd.edu

Nakatsuji T, Liu Y-T, Huang C-P, Zouboulis CC, Gallo RL, et al. (2008) Vaccination Targeting a Surface Sialidase of *P. acnes*: Implication for New Treatment of Acne Vulgaris. PLoS ONE 3(2): e1551. doi:10.1371/journal.pone.0001551 

