# Correction: Vaccination Targeting a Surface Sialidase of *P. acnes:* Implication for New Treatment of Acne Vulgaris

**DOI:** 10.1371/annotation/8466fafc-c477-4b2e-b781-97223fb05203

**Published:** 2008-08-19

**Authors:** Teruaki Nakatsuji, Yu-Tsueng Liu, Cheng-Po Huang, Christos C. Zouboulis, Richard L. Gallo, Chun-Ming Huang

Because the author Christos C. Zouboulis was added to the author byline after publication, the Author Contributions section does not correctly reflect the new authorship. There should be an additional section which reads: Contributed reagents/materials/analysis tools: CCZ

