# Supplementary figures and images for: Vaccination Targeting a Surface Sialidase of P. acnes: Implication for New Treatment of Acne Vulgaris
Source: PLoS One. 2008 Feb 6;3(2):e1551. doi: 10.1371/journal.pone.0001551 (PMC2212713; doi:10.1371/journal.pone.0001551)

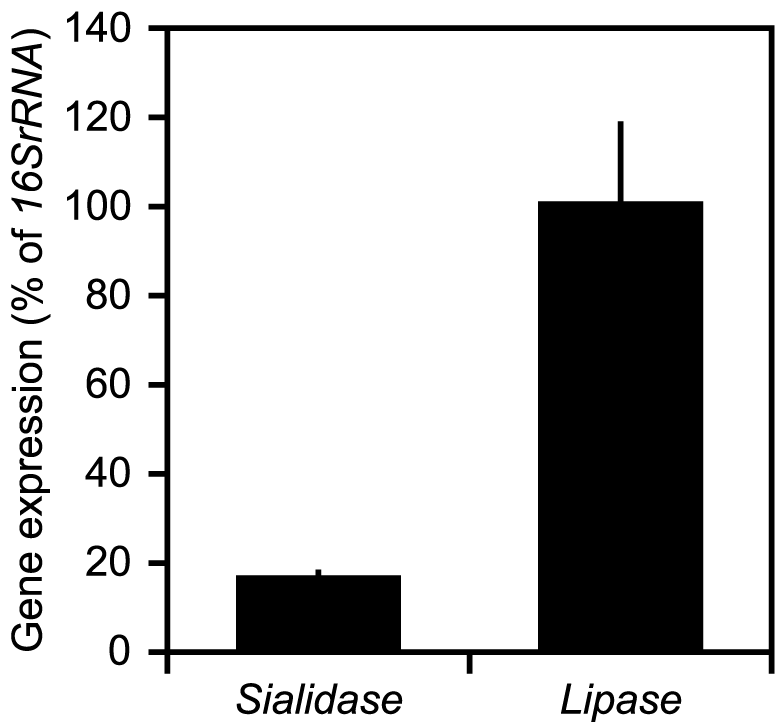

Supplement: Figure S1 — Quantitative analysis of the sialidase transcript in P. acnes. The gene expression of sialidase was determined by real-time quantitative PCR using specific primers as described in Methods. Total RNA isolated from anaerobically cultured P. acnes served as a template. The gene of triacylglycerol lipase known as a pathogenic factor of P. acnes was used as a positive control. A pGEM-T Easy Vector (Promega, Madison, WI) inserted with PCR products was performed to estimate the number of expressed genes. The level of gene expression of sialidase and triacylglycerol lipase was normalized to that of 16SrRNA gene. (0.56 MB TIF) [file pone.0001551.s001.tif]

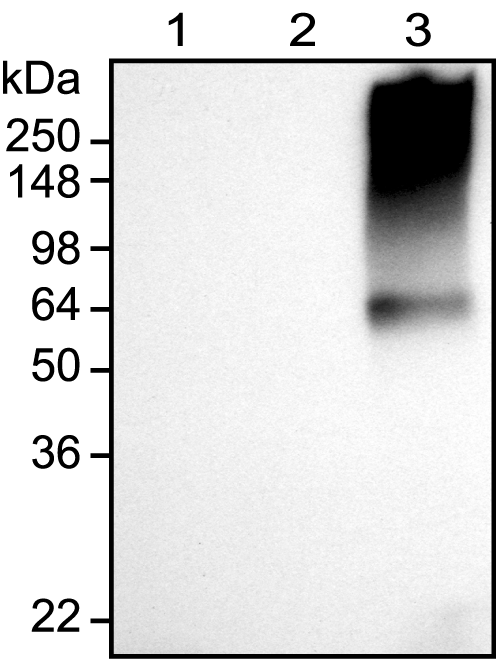

Supplement: Figure S2 — Detection of immunogenicity of sialidase in mice vaccinated with UV-killed P. acnes. ICR mice were vaccinated with UV-killed P. acnes as described in Methods. Serum (1: 500 dilution) was reacted to recombinant sialidase (1 µg; lane 1), GFP (1 µg, lane 2), and P. acnes lysates (7 µg, lane 3) that had been run on a 10% SDS-PAGE. Sialidase and GFP were not immunoreactive to serum obtained from mice immunized with UV-killed P. acnes. (0.33 MB TIF) [file pone.0001551.s002.tif]
