# Supplementary material for: Vaccination Targeting a Surface Sialidase of P. acnes: Implication for New Treatment of Acne Vulgaris
Source: PLoS One. 2008 Feb 6;3(2):e1551. doi: 10.1371/journal.pone.0001551 (PMC2212713; doi:10.1371/journal.pone.0001551)
Supplement: Text S1 — (0.02 MB DOC) [file pone.0001551.s003.doc]

**SUPPORTING INFORMATION**

**Text S1**

**Quantification of sialidase expression by real-time PCR**

Total RNA was extracted from *P. acnes* using TRIzol Max Bacterial RNA Isolation Kit (Invitrogen, Carlsbad, CA) and the first strand cDNA was synthesized using mixture of gene specific primers for sialidase (5’-ATAGGAAGGATCGGAGTAT-3’), triacylglycerol glycerol lipase (accession number: gi|50841145) (5’-ACGGGATCATAGGGTAG-3’), and 16SrRNA (5’-GTGCTTCTTTACCCATTAC-3’) with SuperScript III (Invitrogen, Carlsbad, CA). The gene expression of sialidase was analyzed by a quantitative real-time PCR via an ABI PRISM 7000 (ABI, Drive Foster City, CA). The forward primers for sialidase and triacylglycerol lipase were 5’-CCAAGGGAGCCATTATGAAA-3’ and 5’-GGATTTCCTTAGCACGTGGA-3’, respectively. The reverse primers [5’-TGCTCATTGACAGCCAAATC-3’ (sialidase) and 5’-GGGTGGAGATGACGGTGTAG-3’ (triacylglycerol lipase)] were designed. PCR reaction was performed with SYBR Green PCR Master MIX (ABI, Foster City,, CA). The program consisted of an initial denaturation (95 ^o^C, 3 min) and followed by 35 cycles of amplification (94 ^o^C, 15 s; 55 ^o^C, 15 s; 72 ^o^C, 30 s). The melting curve analysis was used to examine the specificity of the PCR product. Serial 1: 10 dilutions of a pGEM-T Easy Vector (Promega, Madison, WI) containing the PCR product inserts (10^3^-10^8^ copies/ assay) were performed to estimate the number of expressed genes. Potential genomic DNA contamination was examined by including non-reverse transcribed total RNA as a template. The expression levels of sialidase and triacylglycerol lipase genes were normalized to the expression level of 16SrRNA (accession number: gi| 50841496). The forward and reverse primers for 16SrRNA were 5’-TGGGGTAGTGGCTTACCAAG-3’, and 5’-GTGCAATATTCCCCACTGCT-3’, respectively.
